# Distribution of PLGA-modified nanoparticles in 3D cell culture models of hypo-vascularized tumor tissue

**DOI:** 10.1186/s12951-017-0298-x

**Published:** 2017-10-05

**Authors:** Lee B. Sims, Maya K. Huss, Hermann B. Frieboes, Jill M. Steinbach-Rankins

**Affiliations:** 1 Department of Bioengineering, University of Louisville, 505 S. Hancock, CTRB 623, Louisville, KY 40208 USA; 2 James Graham Brown Cancer Center, University of Louisville, Louisville, KY USA; 3 Department of Pharmacology and Toxicology, University of Louisville, Louisville, KY USA; 4 Department of Microbiology and Immunology, University of Louisville, Louisville, KY USA; 5 Center for Predictive Medicine, University of Louisville, Louisville, KY USA

**Keywords:** Nanoparticles, Cell penetrating peptide (CPP), Cervical cancer, Nanoparticle transport, Tumor vascularization, 3D cell culture, Tumor spheroid

## Abstract

**Background:**

Advanced stage cancer treatments are often invasive and painful—typically comprised of surgery, chemotherapy, and/or radiation treatment. Low transport efficiency during systemic chemotherapy may require high chemotherapeutic doses to effectively target cancerous tissue, resulting in systemic toxicity. Nanotherapeutic platforms have been proposed as an alternative to more safely and effectively deliver therapeutic agents directly to tumor sites. However, cellular internalization and tumor penetration are often diametrically opposed, with limited access to tumor regions distal from vasculature, due to irregular tissue morphologies. To address these transport challenges, nanoparticles (NPs) are often surface-modified with ligands to enhance transport and longevity after localized or systemic administration. Here, we evaluate stealth polyethylene–glycol (PEG), cell-penetrating (MPG), and CPP-stealth (MPG/PEG) poly(lactic-*co*-glycolic-acid) (PLGA) NP co-treatment strategies in 3D cell culture representing hypo-vascularized tissue.

**Results:**

Smaller, more regularly-shaped avascular tissue was generated using the hanging drop (HD) method, while more irregularly-shaped masses were formed with the liquid overlay (LO) technique. To compare NP distribution differences within the same type of tissue as a function of different cancer types, we selected HeLa, cervical epithelial adenocarcinoma cells; CaSki, cervical epidermoid carcinoma cells; and SiHa, grade II cervical squamous cell carcinoma cells. In HD tumors, enhanced distribution relative to unmodified NPs was measured for MPG and PEG NPs in HeLa, and for all modified NPs in SiHa spheroids. In LO tumors, the greatest distribution was observed for MPG and MPG/PEG NPs in HeLa, and for PEG and MPG/PEG NPs in SiHa spheroids.

**Conclusions:**

Pre-clinical evaluation of PLGA-modified NP distribution into hypo-vascularized tumor tissue may benefit from considering tissue morphology in addition to cancer type.

**Electronic supplementary material:**

The online version of this article (doi:10.1186/s12951-017-0298-x) contains supplementary material, which is available to authorized users.

## Background

Relative to effective and non-invasive preventative options such as vaccines, late-stage cancer treatments are usually invasive and painful, and typically include surgery, chemotherapy, and radiation treatment. Chemotherapy often induces irreversible damage to surrounding healthy tissue as well as incomplete tumor eradication. For systemic chemotherapy specifically, it can be challenging to achieve distribution throughout the tumor to maximize treatment effectiveness. Nanotherapeutic platforms have been proposed as safer and more effective modalities to deliver therapeutic agents directly to the tumor site. In particular, FDA-approved polymer-based platforms such as poly(lactic-*co*-glycolic) acid (PLGA) NPs, have been utilized to reduce unwanted immunogenic responses. Although NPs have been surface-modified with a variety of ligands to enhance tumor penetration and targeting [1–9], currently, two delivery paradigms exist, often with cellular internalization and tissue penetration diametrically opposed. In trying to achieve enhanced cellular internalization, the efficacy benefit may be limited if surface-modification prevents the carrier from penetrating deeply into the tumor interstitium. Conversely, if penetration into the tumor interstitium is successfully achieved—thereby providing broad distribution throughout the tumor—delivery vehicles may be inadequately internalized by the cells targeted. Unfortunately, similar ineffective therapy results in both cases.

To balance these transport challenges, NPs are often surface-modified with ligands to enhance transport and longevity after localized or systemic administration. One of the most common ligands used to functionalize and promote NP delivery, poly(ethylene-glycol) (PEG), has been employed as a “stealth” modification, due to its hydrophilic and easily tailorable properties. PEG has been shown to increase vehicle circulation time by decreasing unwanted systemic interactions, and has enhanced transport through interstitial space and intercellular junctions [9–18]. In contrast, cell penetrating peptides (CPPs)—short amphipathic or polycationic peptides—have been utilized to improve the intracellular delivery of cargo. Due to their cationic and sometimes lipophilic properties, CPPs have been designed to promote the internalization of attached cargo across cell membranes, particularly for gene delivery applications [2, 4, 6, 8, 13, 19, 20].

For cervical cancer specific applications, a variety of polymeric NP formulations have been recently investigated to deliver chemotherapeutics. Nanoparticle derivatives of PLGA [7, 21–24] have demonstrated sustained delivery of docetaxel against cervical cancer both in vitro and in vivo, correlated with high uptake and corresponding antitumor effects. Similarly, Eudragit-E and polyvinyl alcohol NPs containing Naringenin induced dose-dependent cytotoxicity [25]. In another study, genistein-encapsulated ε-caprolactone-based NPs demonstrated enhanced cytotoxicity and growth inhibition in a murine HeLa xenograft tumor model [26]. Folate-targeted doxorubicin-loaded NPs have improved targeting and anti-tumor efficacy in vivo [18] and pullulan acetate folate-modified NPs were used to treat cervical carcinoma [27]. Peng et al. utilized a thermosensitive gel to target DNA poly(β-amino ester) NPs to ex vivo pre-neoplastic cervical lesions and mouse cervical tissue [28]. Similarly, Blum et al. developed topical camptothecin-loaded PLGA nanoparticles to prevent tumor growth in an inducible murine model of vaginal squamous cell carcinoma [29]. Most recently, Yang et al. evaluated paclitaxel mucus penetrating or adhesive PLGA NPs, demonstrating significantly less tumor growth and increased survival with mucoadhesive NPs [30].

Recently, we investigated the effects of NP surface-modification with cell-penetrating peptides (CPPs), stealth ligands, and tumor targeting ligands (MPG (unabbreviated notation), PEG, and Vimentin, respectively), on NP penetration and distribution within the hypo-vascularized tumor environment [15]. Nanoparticles modified with a CPP, MPG, exhibited the highest cellular internalization in human cervical carcinoma (HeLa) 3D cell culture (multi-cellular spheroids); however, internalization primarily occurred within the spheroid periphery, resulting in a modest (< 100 nm) distribution profile. In contrast, PEG-modified NPs distributed more deeply into spheroids, but were less readily internalized by cells. These results seemed to indicate that tissue morphology in addition to NP functionalization were key factors determining NP distribution.

**Fig. 1:**
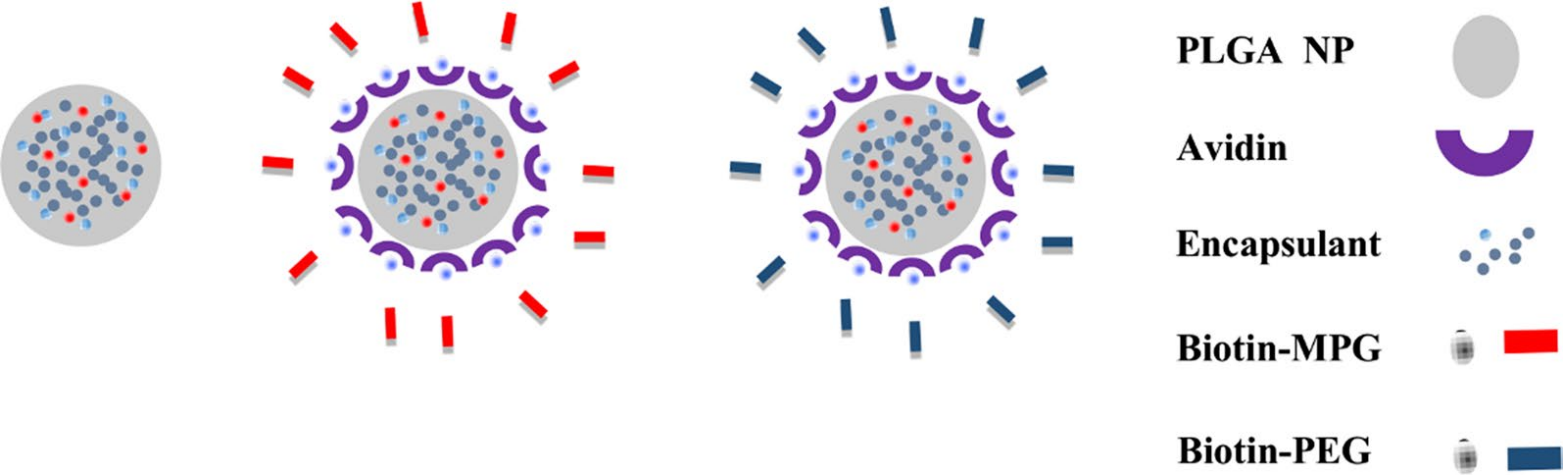
Schematic representing NP formulations used in this study. From left to right: unmodified, MPG, and PEG formulations

In this study, we consider tissue morphology while evaluating the penetration and distribution of PEG, cell penetrating (MPG), and CPP-stealth (MPG/PEG) NP co-treatment strategies in models of hypo-vascularized cervical cancer tissue representing regions distal from the point of vascular extravasation (Fig. 1). To model more regularly-shaped tissue, we utilized the hanging drop (HD) method to generate smaller, more homogeneously spherically-shaped spheroids. We compared NP penetration to that in tissue formed via the liquid overlay (LO) technique, in which spheroids form more irregularly-shaped masses. To compare differences in NP distribution within the same type of lesion but as a function of different cervical cancer types, we selected HeLa, cervical epithelial adenocarcinoma cells; CaSki, cervical epidermoid carcinoma cells; and SiHa, grade II cervical squamous cell carcinoma cells. Thus, the goal was to assess differences in NP distribution as a function of NP surface-modification (unmodified, MPG, PEG, or MPG/PEG NP co-treatment), hypo-vascularized tissue (regular or irregular morphology), and cancer cell type.

## Results

**Fig. 2:**
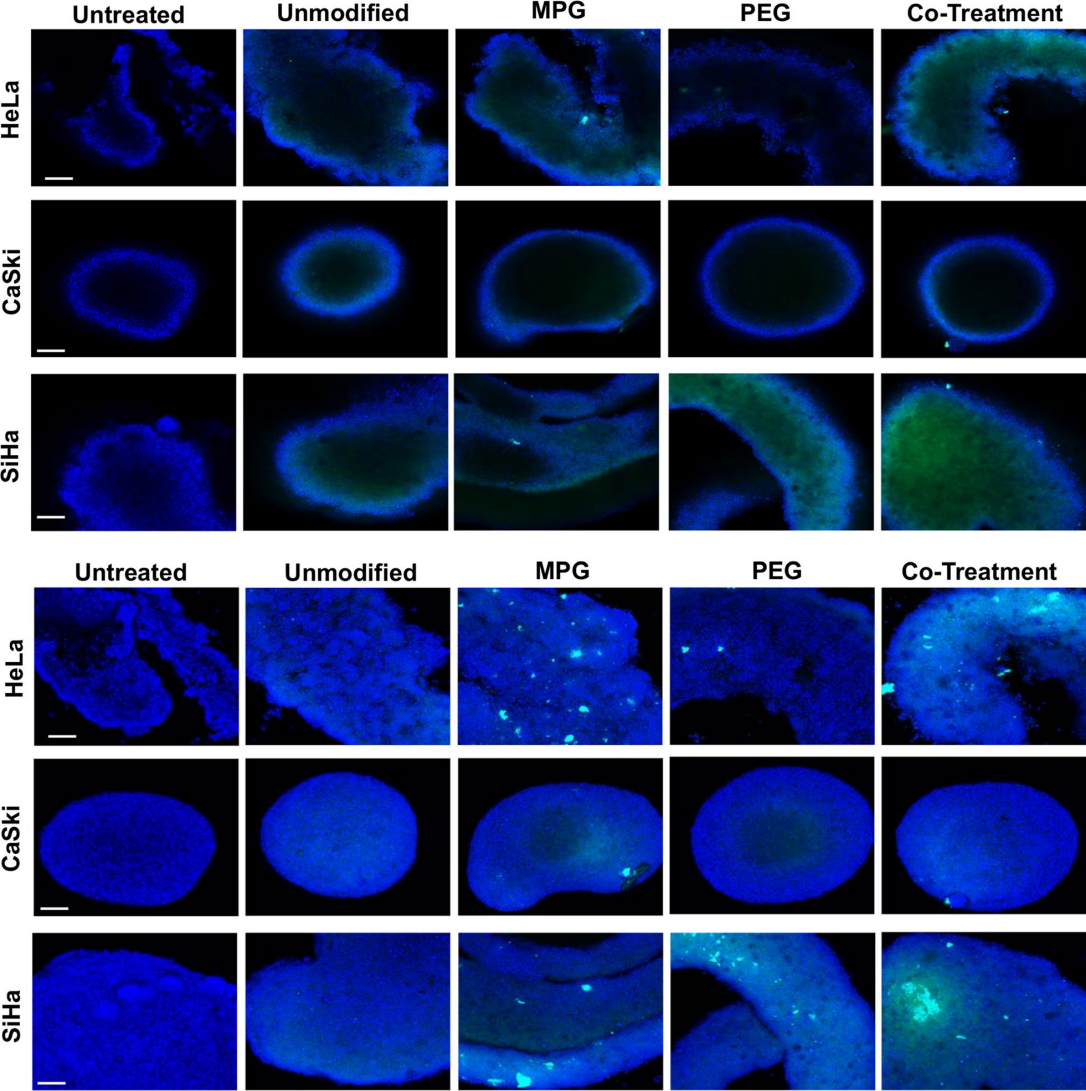
NP distribution through liquid overlay (LO) spheroids in mid-plane cross-sections (top three rows) and 3D composite (bottom three rows) confocal images. Nuclei are blue (Hoechst) and NPs are green (Coumarin 6). Scale bar: 50 μm

**Fig. 3:**
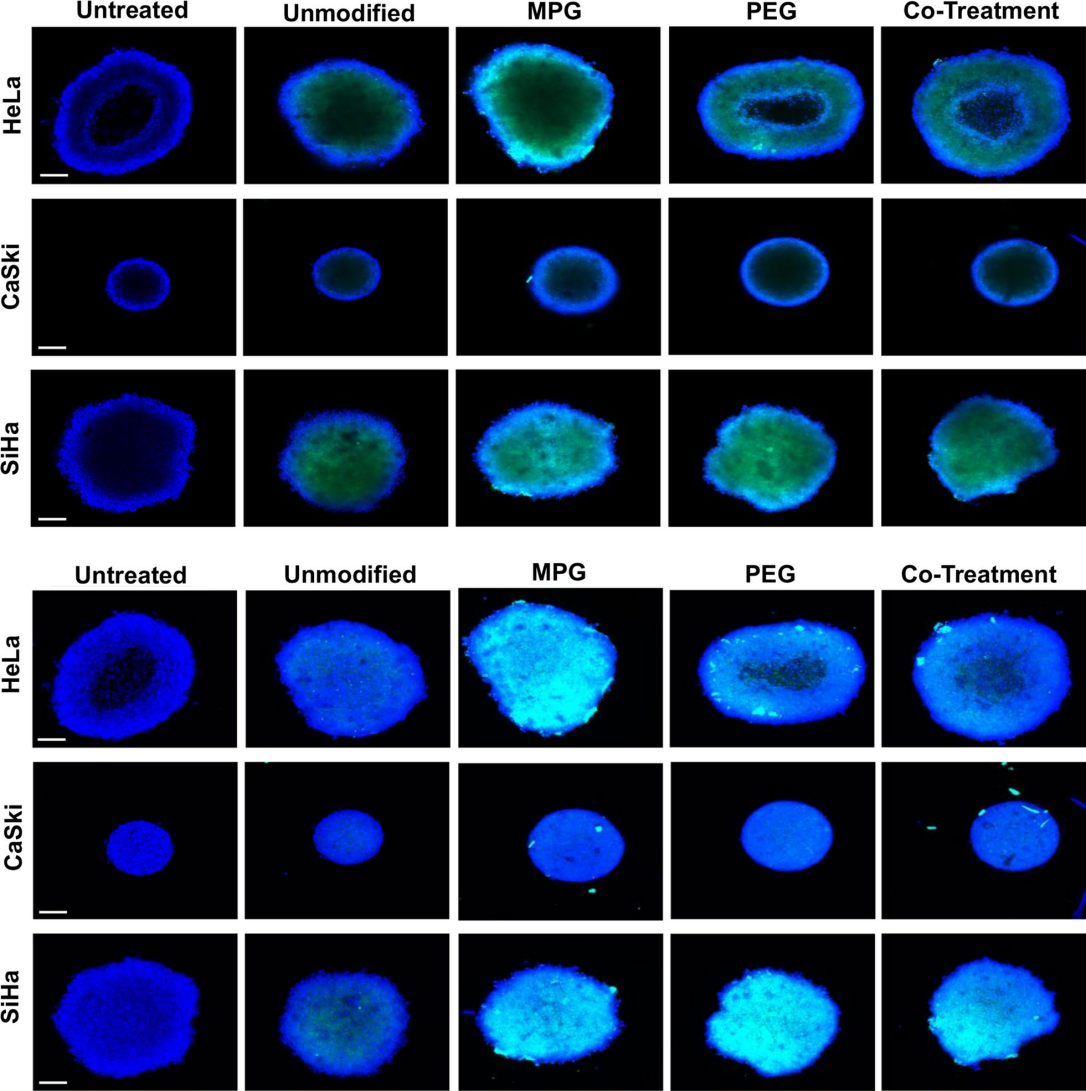
NP distribution through hanging drop (HD) spheroids in mid-plane cross-sections (top three rows) and 3D composite (bottom three rows) confocal images. Nuclei are blue (Hoechst) and NPs are green (Coumarin 6). Scale bar: 50 μm

To assess the distribution of unmodified, PEG, MPG, or MPG/PEG co-treatment NPs into hypo-vascularized cervical cancer tissue regions distal from the point of vascular extravasation, NP diffusion was measured in SiHa, CaSki, or HeLa LO or HD spheroids. The area under the curve (AUC, MFI-μm) was determined, corresponding to the fluorescence intensity of NPs distributed throughout the tumor spheroid, in addition to the maximum mean fluorescence intensity (MFI) observed at a given penetration distance for each spheroid and cell type. The maximum MFI defines the spheroid depth at which the highest MFI was observed within the tumor spheroid after 1.5 h administration. NP penetration and distribution through the spheroids varied as a function of surface modification, tissue morphology (LO or HD), and cell type (Figs. 2, 3).

### Spheroid characterization

In general, the HD spheroids were smaller in size for HeLa, CaSki, and SiHa cells (with respective average maximum cross-sectional areas of 0.31, 0.10, and 0.25 mm^2^) compared to the LO spheroids (with respective areas of 0.55, 0.39, and 0.57 mm^2^)—see Table 1. In addition, for both HD and LO spheroids, CaSki tumors demonstrated statistically significant decreases in size, relative to HeLa or SiHa spheroids.

**Table 1:** Maximum cross-sectional areas of hanging drop (HD) and liquid overlay (LO) tumor spheroids as a function of cell type

Spheroid cross-sectional area (mm^2^)		
Cell line	Hanging drop	Liquid overlay
HeLa	0.31 ± 0.02	0.55 ± 0.03
CaSki	0.10 ± 0.02	0.39 ± 0.04
SiHa	0.25 ± 0.03	0.57 ± 0.04

All values represent the average ± standard deviation (n = 3)

### NP characterization

NP size and morphology were confirmed using SEM imaging and ImageJ processing. Unhydrated NPs demonstrated a spherical morphology, with diameters measuring 160 to180 nm [15]. Hydrated NP surface charges were measured using a Zetasizer (Malvern). Unmodified NPs had a negative surface charge of − 26.63 ± 1.05 mV; while PEG- and MPG-modified NPs measured − 22.03 ± 1.40 and − 8.54 ± 0.35 mV, respectively (Table 2), validating surface ligand conjugation.

**Table 2:** Zeta potentials of unmodified, PEG-, and MPG-modified NPs

NP type	NP zeta-potential (mV)
Unmodified	− 26.63 ± 1.05
PEG	− 22.03 ± 1.40
MPG	− 8.54 ± 0.35

Zeta potential values are shown as the average ± standard deviation (n = 3)

### Unmodified NP penetration and distribution

**Fig. 4:**
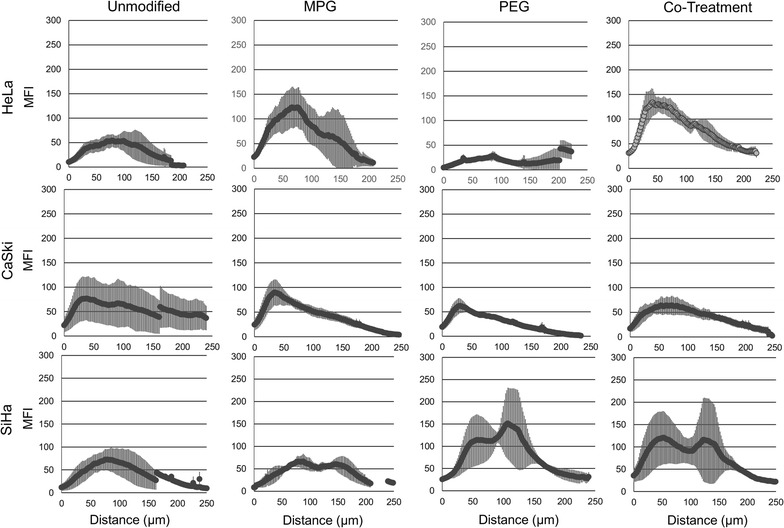
NP distribution profiles quantifying the mean fluorescence intensity (MFI) vs. penetration distance through liquid overlay (LO) spheroids. Distribution profiles are shown as a function of NP treatment and tumor cell type. Average of the values along distance is denoted by the dark lines

**Fig. 5:**
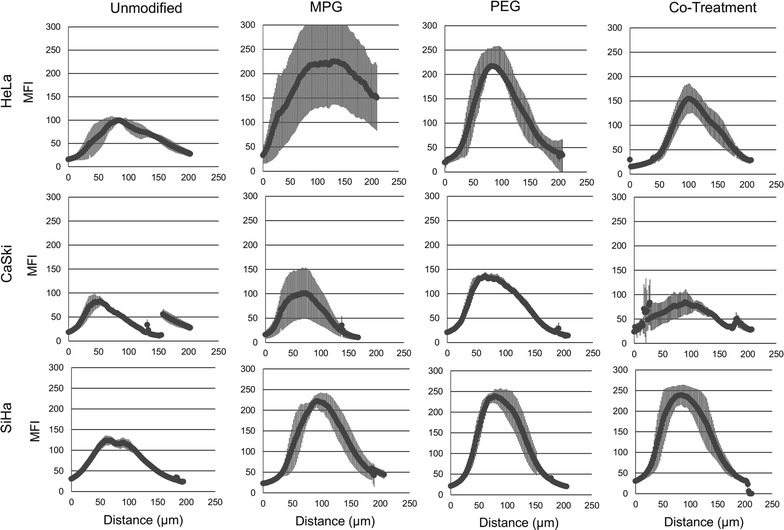
NP distribution profiles quantifying the mean fluorescence intensity (MFI) vs. penetration distance through hanging drop (HD) spheroids. Distribution profiles are shown as a function of NP treatment and tumor cell type. Average of the values along distance is denoted by the dark lines

**Fig. 6:**
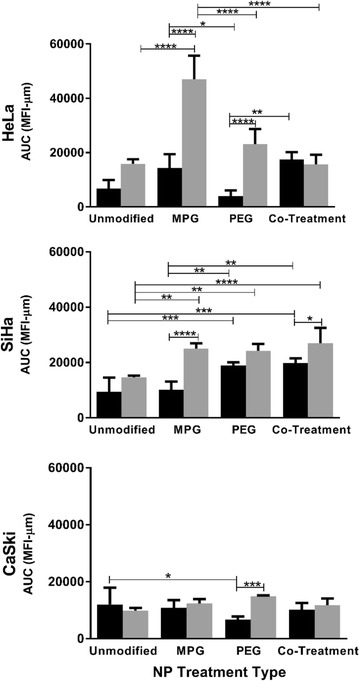
NP distribution represented as AUC for each tumor cell type (HeLa, SiHa, or CaSki) as a function of NP treatment group, relative to spheroid type (LO, black and HD, gray). Values of all significant correlations, including each treatment group relative to unmodified NPs, relative to other treatment groups, or relative to the same treatment group in a different spheroid type are given with degree of significance indicated (* p < 0.01, ** p < 0.001, *** p < 0.0001, **** p < 0.00001). Error bars: average ± standard deviation (n = 3)

NP distribution as a function of cell type and NP formulation was quantified for LO (Fig. 4) and HD (Fig. 5) spheroids, respectively. The AUC as a function of NP treatment group and cell type are plotted in Fig. 6 and Additional file 1: Figure S1, respectively. Table 3 provides the actual AUC values for each treatment and cell type. Despite the minimal distribution observed with unmodified NPs, a statistically significant difference was observed between regularly-shaped (HD) and irregularly-shaped (LO) HeLa spheroids (AUCs LO 6827 ± 3101 and HD 15,841 ± 1637 MFI-μm), while no statistical differences were observed between regularly- and irregularly-shaped CaSki or SiHa cell spheroids. Furthermore, unmodified NPs exhibited similar distribution trends within HeLa, CaSki, and SiHa spheroids of each type (Figs. 4, 5, 6). Within all spheroids, the distribution profiles showed that the maximum MFI occurred within 100 μm of the tumor periphery. Moreover, only SiHa HD spheroids exhibit a maximum MFI peak above 100 MFI (Fig. 5 and Table 4), indicating substantial accumulation of NPs at a given penetration distance. Overall, unmodified NPs show no differential penetration and distribution between tumor/cell types and exhibited only modest distribution within spheroids, relative to surface-modified NP groups.

**Table 3:** NP distribution represented as area-under-the-curve (AUC) (MFI-μm) for each spheroid (LO or HD) and tumor cell type, relative to NP treatment group

Area under the curve (AUC)				
Cell line	Unmodified NPs	MPG NPs	PEG NPs	Co-treatment NPs
HeLa	LO [6827 ± 3101] HD [15,841 ± 1637]	LO [14,339 ± 5129] HD [47,018 ± 8754]	LO [4042 ± 2101] HD [23,140 ± 5531]	LO [17,536 ± 2675] HD [15,657 ± 3579]
CaSki	LO [12,043 ± 5846] HD [9331 ± 1090]	LO [10,847 ± 2673] HD [12,364 ± 1485]	LO [6718 ± 1065] HD [14,297 ± 281]	LO [10,138 ± 2413] HD [11,847 ± 2231]
SiHa	LO [9377 ± 5219] HD [14,673 ± 543]	LO [10,186 ± 2887] HD [24,972 ± 2020]	LO [18,972 ± 1065] HD [24,237 ± 2532]	LO [19,783 ± 1685] HD [26,970 ± 5574]

All AUC values represent the average ± standard deviation (n = 3). These data along with statistical significance are presented in Fig. 6 and Additional file 1: Figure S1

**Table 4:** NP distribution in terms of maximum mean fluorescence intensity (MFI) observed at a given penetration depth (μm) for each spheroid (LO or HD) and tumor cell type, relative to NP treatment group

Maximum penetration peak (MFI, μm)				
Cell line	Unmodified NPs	MPG NPs	PEG NPs	Co-treatment NPs
HeLa	LO (54, 78) HD (99, 85)	LO (123, 76) HD (225, 129)	LO (42, 203) HD (217, 82)	LO (133, 150) HD (154, 102)
CaSki	LO (76, 38) HD (83, 52)	LO (89, 35) HD (101, 66)	LO (62, 39) HD (135, 64)	LO (64, 73) HD (84, 90)
SiHa	LO (72, 79) HD (123, 61)	LO (66, 88) HD (222, 91)	LO (151, 107) HD (238, 78)	LO (120, 52) HD (239, 84)

### MPG NP penetration and distribution

In contrast to the similar distribution profiles of unmodified NPs within a given spheroid or cell type, significant differences in distribution were observed for MPG NPs as a function of tissue morphology and cell type. In HeLa spheroids, MPG NP distribution (AUC) was found to be statistically significant and three times higher in regularly-shaped (HD) spheroids (47,018 ± 8751 MFI-μm), compared to irregularly-shaped (LO) spheroids (14,339 ± 5129 MFI-μm). Similarly, MPG NP distribution was doubled in SiHa regularly-shaped spheroids relative to SiHa irregularly-shaped spheroids (AUCs 24,972 ± 2020 and 10,186 ± 2887 MFI-μm). However, a minimal difference in MPG NP distribution was observed between CaSki spheroids (Figs. 4, 5, 6, Table 3). Overall, MPG NPs distributed significantly more in HeLa spheroids (47,018 and 14,339 MFI-μm), relative to the other cell types.

Additionally, MPG NPs provided much greater intratumoral accumulation when compared to unmodified NPs, and the distribution trends varied as a function of cell type. In regularly-shaped HD spheroids, MPG NPs penetrated deeper, and accumulated in greater amounts (represented by increased MFI) relative to irregularly-shaped LO spheroids. In HeLa regularly-shaped spheroids, NPs exhibited the greatest peak in accumulation 129 μm (225 MFI) from the tumor periphery, with the second highest peak observed in SiHa cells (222 MFI) 91 μm from the periphery (Fig. 5 and Table 4). Similar to irregularly-shaped spheroids, NP distribution through regularly-shaped CaSki spheroids was dampened by comparison.

In irregularly-shaped LO HeLa spheroids, MPG NPs demonstrated peaks 76 μm (123 MFI) and 133 μm (63 MFI) from the periphery (Fig. 4, Table 4). These values were significantly higher than exhibited by MPG NPs in irregularly-shaped LO SiHa (MFI 66) or CaSki (MFI 89) tumors. In contrast to these trends, CaSki cells demonstrated gentler and more uniform distribution curves, with less pronounced maximum MFIs, similar to unmodified NPs.

Overall, MPG NPs exhibited increased distribution in the smaller regularly-shaped spheroids relative to larger, irregularly-shaped spheroids, across all cell lines. HeLa and SiHa regularly-shaped spheroids seemed to offer the most permissive environments to MPG NP intratumoral distribution and accumulation.

### PEG NP penetration and distribution

We observed that NPs modified with PEG exhibited variations in tumor penetration and distribution as a function of both cell and tissue type. In the larger, irregularly-shaped spheroids, PEG NPs had the greatest intratumoral distribution in SiHa cells (AUC 18,972 ± 1065 MFI-μm) and exhibited a bimodal distribution trend, relative to the dampened distribution observed in irregularly-shaped HeLa and CaSki spheroids (AUCs 4042 ± 2101 and 6718 ± 1065 MFI-μm) (Figs. 4, 5, 6, Table 3). In contrast, significantly enhanced distribution of PEG NPs was observed within the regularly-shaped HD spheroids, with statistically significant increases in PEG NP distribution observed in HeLa and SiHa, relative to CaSki spheroids (AUCs 23,140 ± 5531; 24,237 ± 2532; 14,297 ± 281, respectively). Most notably, PEG NP distribution in regularly-shaped HeLa spheroids was nearly six-fold greater than in the irregularly-shaped tissue (Figs. 4, 5, 6, Table 3).

In regularly-shaped HD spheroids, PEG NPs exhibited similar unimodal distribution trends across all cell lines. SiHa regularly-shaped spheroids showed the greatest NP accumulation (238 MFI) occurring 78 μm from the spheroid periphery (Fig. 5, Table 4). Moreover, NPs penetrated more deeply, and with higher accumulation, into regularly-shaped HeLa and SiHa spheroids (82 and 78 μm), relative to regularly-shaped CaSki spheroids (64 μm). In contrast, PEG NP distribution through the irregularly-shaped spheroids varied significantly as a function of cell type (Fig. 4, Table 4). In irregularly-shaped LO CaSki spheroids, the observed distribution profile was quite dampened, exhibiting a peak of maximum accumulation (62 MFI) only 39 μm from the tumor periphery, followed by a decrease in penetration. In comparison, irregularly-shaped SiHa spheroids exhibited a bimodal distribution of PEG NPs, with minor and major maximum peaks (114 and 151 MFI) occurring 62 and 107 μm from the spheroid periphery. In contrast, a maximum accumulation of only 42 MFI was reached 203 μm from the spheroid periphery for HeLa cells (Fig. 4, Table 4). Overall, PEG NPs exhibited significant penetration and distribution in SiHa regularly- as well as irregularly-shaped spheroids, with a maximum intratumoral distribution observed in the regularly-shaped HeLa case.

### MPG/PEG co-treatment penetration and distribution

The MPG/PEG NP co-treatment strategy also demonstrated differences in NP distribution as a function of tumor and cell type, similar to other modified NP groups. In regularly-shaped spheroids, the co-treatment groups demonstrated the greatest distribution in SiHa cells (AUC 26,970 ± 5574 MFI-μm) (Figs. 5, 6, and Table 3). Similarly, the MPG/PEG co-treatment NPs distributed well in HeLa regularly-shaped spheroids (AUC 15,657 ± 3579 MFI-μm), although there was no statistical significance observed in the AUC relative to regularly-shaped CaSki cells (Figs. 5, 6, and Table 3). In both CaSki spheroid types, the NP co-treatment group exhibited statistically decreased levels of distribution relative to that observed in HeLa and SiHa cells. In addition, for irregularly-shaped spheroids, insignificant differences in NP distribution were observed between HeLa and SiHa cells (AUCs 17,536 ± 2675 and 19,783 ± 1685 MFI-μm), although the distribution trend differed between cell lines (Figs. 4, 6, Table 3). Overall, the MPG/PEG co-treatment had the strongest distribution trends (> 15,000 MFI-μm) in the HeLa and SiHa spheroids, regardless of tumor type.

In terms of accumulation and penetration, within the regularly-shaped spheroids (Fig. 5, Table 4), the MPG/PEG NP co-treatment group exhibited the highest accumulation in SiHa cells (maximum peak 239 MFI), relative to that observed in HeLa (154 MFI) and CaSki cells (84 MFI). However, MPG/PEG NPs exhibited increased penetration in HeLa and CaSki regularly-shaped spheroids (102 μm and 90 μm, respectively). Additionally, SiHa and HeLa spheroids exhibited sharp unimodal peaks, whereas a more diminished distribution was observed in CaSki spheroids (Fig. 5). In HeLa and CaSki irregularly-shaped spheroids (Fig. 4), the trends were similar as observed in regularly-shaped spheroids, although NPs accumulated and penetrated less deeply in irregularly-shaped spheroids and exhibited more modest distribution. Overall, the MPG/PEG co-treatment NPs demonstrated the greatest distribution and penetration in SiHa and HeLa spheroids representing regularly- and irregularly-shaped avascular tissue.

## Discussion

We have previously evaluated the effect of MPG, PEG, tumor-targeting (VIM), and hybrid (MPG + PEG) surface-modified PLGA NPs on NP internalization and distribution in HeLa cell monolayers and 3D LO spheroids [15]. We found that MPG-modified NPs offered the greatest internalization in monolayers and spheroids, and exhibited the highest intratumoral accumulation. Yet, despite high levels of distribution and internalization, the most effective particle group, MPG NPs, was sequestered within the tumor periphery, while PEG-modified NPs penetrated more deeply into the tumor interstitium. Additionally we noted that surface modification with opposing ligands on the same NP hindered penetration, which we attributed to potential steric hindrance or competing functionalities interacting with the ECM or tumor matrix. To expand upon this work, here we evaluate the impact of tumor size, tissue morphology, and cell origin on NP distribution. To assess these conditions, we used two different spheroid formation methods (LO and HD) to produce 3D spheroids that have been previously shown [31] to respectively represent irregularly- or more regularly-shaped hypo-vascularized tumor regions distal from the location of vascular extravasation. We evaluated three different cervical carcinoma cell lines including HeLa, SiHa, and CaSki cells in both tumor models. Using the information gained from these studies, our goal was to provide insight into NP treatment strategies that may be applied to different types of tumors.

We observed that NPs achieved greater distribution in the smaller regularly-shaped spheroids, accompanied by increased penetration in HeLa and SiHa HD spheroids, relative to the larger, irregularly-shaped LO spheroids. Yet regardless of spheroid formation method, surface-modified NPs consistently exhibited increased penetration and distribution, relative to unmodified NPs. In irregularly-shaped spheroids, PEG and MPG/PEG co-treatment groups demonstrated the greatest distribution in SiHa cells (AUCs 18,972 ± 1065 and 19,783 ± 1685 MFI-μm), followed by MPG and MPG/PEG in HeLa (AUC 14,339 ± 5129 and 17,536 ± 2675 MFI-μm), whereas all NPs demonstrated rather modest penetration in irregularly-shaped CaSki spheroids. In comparison, in regularly-shaped spheroids, MPG exhibited the greatest distribution (47,018 ± 8754) in HeLa cells, with similar modest distribution of all NP groups through CaSki spheroids. In regularly-shaped SiHa spheroids, MPG, PEG, and MPG/PEG groups exhibited similar distribution (24,972 ± 2020, 24,237 ± 2532, and 26,970 ± 5574 MFI-μm). In both regularly- and irregularly-shaped SiHa and HeLa spheroids, we observed only modest distribution of unmodified NPs, relative to other NP groups. In CaSki regularly-shaped spheroids, unmodified NPs demonstrated similar distribution (12,043 ± 5846 MFI-μm) relative to other NP groups.

With these studies in mind, MPG NPs seem to be the most effective treatment group for the regularly-shaped HeLa tissue, whereas MPG, PEG, or MPG/PEG co-treatment may be used interchangeably for the regularly-shaped SiHa tissue. For larger, irregularly-shaped tissue, MPG and MPG/PEG or PEG and MPG/PEG NPs demonstrated promise for HeLa and SiHas respectively. Importantly, the MPG/PEG co-treatment consistently demonstrated NP penetration and distribution across all cell types relative to other NP groups, offering a potentially comparable treatment option relative to individual groups of surface-modified NPs. In future work, the NP co-treatment strategy may offer a significant increase in NP penetration and distribution across all cell and tumor types, if administered at the same individual NP concentrations as single NP treatments.

While the tumor microenvironment presents many challenges to achieving efficacious cargo delivery, our goal in these studies was to employ rational design to develop more therapeutically efficacious drug and gene delivery vehicles to overcome these challenges. One method used to enhance the delivery of active agents to cells is to directly complex or conjugate ligands to enhance tumor internalization and distribution. Notably, CPPs, such as Tat (the HIV transactivator protein) and MPG, have been widely used as drug and oligonucleotide conjugates to significantly enhance cellular internalization and localization [6, 8, 12, 20, 32, 33]. In parallel work, many groups have conjugated ligands to delivery vehicles, such as NPs, to enhance the distribution of larger cargo within and to the tumor microenvironment [11, 15, 20, 34–39].

Another common strategy to enhance NP distribution, has been to modify the NP surface with different densities of, and molecular weight PEG molecules. In addition to reducing unwanted immune response and increasing the systemic half-life of NPs, this “PEGylation” [10, 16, 17, 30] has enabled enhanced NP distribution in normal tissue or mucosal environments such as the female reproductive tract, gastrointestinal tract, and lung airways [3, 9, 10, 16, 17, 20, 30, 40, 41]. Yet despite these contributions, there are few studies that have assessed how differences in tumor tissue morphology and cell origin, in addition to stealth or cell penetrating NP functionalization affect distribution. Our goal was to assess the impact these factors have on NP distribution through hypoxic/avascular regions of the heterogeneously vascularized tumor microenvironment in a cervical carcinoma model. In addition to being distal to vasculature and hence liable to receive less NPs/drugs, these regions are also usually resistant to cell-cycling drugs due to hypoxia-induced cell quiescence. The strategy proposed here is to enable more homogeneous and increased NP distribution into these regions, and for the NPs to remain long enough to affect cells once they resume cycling upon restored access to oxygen/nutrients.

It is well known that spheroid growth impacts cell proliferation, apoptosis, and necrotic/hypoxic core formation (e.g., [42]), with the outer layer (width ~ 100 μm) mostly proliferative, the middle layer hypoxic, and the inner core necrotic, as we have previously observed [43, 44]. These conditions hold true independent of spheroid type [43], as the diffusion of oxygen and nutrients into this system maintains cell populations with varying proliferative capability, contributing to chemoresistance for cell cycle-specific drugs, and replicates the diffusion limitations of blood-borne substances observed for tissue in vivo [45–48]. Accordingly, the bulk of NP uptake is expected to occur in the spheroid outer proliferative regions, as we have previously measured [12]. Applying this knowledge to our studies, we suggest that highly proliferating regions at the tumor periphery, representing tissue adjacent to vasculature, combined with dormant/quiescent cells within the core, representing tissue distal from nanoparticle point of vascular extravasation, may form a gradient in the larger, irregularly-shaped (LO in our studies) tumor microenvironment, which may impede NP distribution [49]. Therefore we propose that the smaller HD spheroids, more closely mimicking regularly-shaped tumor tissue, likely have less structural variability and less necrotic tissue, perhaps contributing to the more uniform and increased distribution profiles observed.

In addition to tumor size and morphology, we observed that NP distribution varied as a function of cell type. Indeed, likely as important as tumor size, cell origin plays a significant role in the development and diversity of the tumor microenvironment. Overall, we observed that both regularly and irregularly-shaped CaSki spheroid types seem to enable significantly less NP transport across all treatment groups when compared to SiHa and HeLa spheroids. We postulate that this difference in distribution may be due to ability of CaSki cells to form tighter intercellular junctions [50], resulting in more regularly-shaped tissue with an intricate interstitial microenvironment. In such an environment, we may expect NP distribution to be hindered relative to less connected tissue. Further experimental investigation of junctional complex expression or integrity will be pursued in follow-up work, and is expected to provide more detailed information for optimizing targeting strategies. Furthermore, it is interesting to note that the MPG/PEG co-treatment NPs had consistently high NP distribution across all cell and spheroid types, particularly relative to other NP treatment groups in a given cell type. This suggests that the dual delivery strategy of MPG/PEG modified NPs is an efficacious treatment to penetrate and distribute throughout cervical tumors of different cell origins and varying degrees of tissue morphology.

One factor that may lead to similarities and differences seen between these different cell types is that they each possess HPV genomes of different subtypes. Other groups have studied immortalized cell lines derived from different tumor subtypes within the female reproductive tract to better understand phenotypic expression [51–53]. Additionally, the impact of incorporated HPV genomes on protein expression within 3D environments has been evaluated. It was discovered that HeLa cells, derived from an adenocarcinoma tumor subtype of the cervix, contain approximately 20–50 copies of integrated HPV-18, whereas SiHa cells, derived from grade II squamous cell carcinoma, and CaSki cells, derived from cervical epidermoid carcinomas, contain approximately 1–2 and 500 copies of integrated HPV-16, respectively [51]. Previous studies investigating the relationship between gap junctions, connexins, and tumor invasion as a function of HPV-related cervical cancer progression found that all three HPV-associated cervical cancer cell lines were poorly coupled and formed no appreciable gap junctions. Furthermore, both SiHa and HeLa cells were observed to have negligible levels of Connexin43 (an important transmembrane protein responsible for gap junction assembly), while very low levels were expressed in CaSki cells [52]. These findings support our observations that SiHa and HeLa cells are more permissive to transport than CaSki spheroids possibly due to these varying levels of gap junction proteins. Additionally, CaSki cells consistently formed more regularly-shaped spheroids, independent of spheroid formation method (HD or LO), indicating the impact of cell phenotype on tumor morphology.

In a separate study, the tumorigenicity of these cell lines was evaluated by observing the tumor forming ability post-injection in athymic mice; SiHa and HeLa cells were found to form tumors at lower cell density injections relative to CaSki cells. In the same study, SiHa and HeLa cells were shown to upregulate cancer-inducing cell-specific genes such as stem-cell markers, integrins, and epithelial to mesenchymal transition associated genes, and were found to be highly tumorigenic [53]. Taking these studies into consideration, it is likely that tumor spheroids formed from different cervical carcinoma cell lines generate varying tumor microenvironments and, therefore, affect NP penetration and distribution. Furthermore, it seems that capitalizing on the properties of a cationic CPP and neutral, hydrophilic ligand modified NPs may result in increased NP distribution through and accumulation in the tumor microenvironment, as shown here (Figs. 4, 5; Tables 3, 4).

## Conclusions

We have presented single and dual-delivery strategies, with NPs modified with opposing stealth and cell penetration ligands, targeting hypovascularized tumor tissue. The results suggest that the co-treatment delivery strategy may provide the greatest intratumoral delivery and diffusion across a variety of cervical cancer disease/cell origins. When considering NP treatment for more regularly-shaped tissue, all surface-modified groups had similar distribution trends, relative to unmodified NPs. Moreover, for both regularly- and irregularly-shaped tumors, if MPG/PEG NPs are administered at the same concentration as the modified NPs alone, the co-treatment has the potential to offer an increase in NP penetration and distribution in all cell and tumor types. This provides hope that a NP co-treatment strategy may overcome the obstacles associated with NP tumor penetration and distribution and may influence the design of delivery platforms for cancer therapy, especially since tumors are expected to present both regularly- and irregularly-shaped tissue regions. Longer term, the knowledge gained from these studies may offer guidance regarding the most efficacious treatment strategies to treat tumors of varying disease type origins and stages of progression.

## Methods

### Synthesis of avidin-palmitate conjugates

Avidin-palmitate was synthesized for subsequent conjugation to NPs as previously described [15, 20, 40]. Briefly, 40 mg of avidin (A9275, Sigma) was dissolved in 4.8 mL of 2% sodium deoxycholate (NaDC) in phosphate buffered saline (PBS) warmed to 37 °C. Palmitic acid-NHS (PA-NHS, Sigma) was dissolved in 2% NaDC to a final concentration of 1 mg/mL and sonicated until well-mixed. PA-NHS solution (3.2 mL) was added dropwise to the avidin NaDC solution, and reacted overnight at 37 °C. The following day, the reaction was dialyzed in 1200 mL of 0.15% NaDC in PBS heated to 37 °C. Free PA-NHS was dialyzed overnight at 37 °C using 3500 MWCO tubing to remove free palmitic acid. After overnight dialysis, the dialysis tubing contents were transferred to a storage vial and stored at 4 °C until use.

### Nanoparticle synthesis

PLGA NPs encapsulating the fluorophore Coumarin 6 (C6) were synthesized as previously described [15, 41] to enable visualization via fluorescence microscopy. From earlier studies [54–57], as well as our previous experiments [15, 20], we have observed that negligible quantities (~ 1%) of C6 are released from NPs. This is attributed to the hydrophobic nature of C6 encapsulated within hydrophobic NPs. Therefore, C6 detected in cells reflects NP distribution in or bound to the cells, not C6 release and distribution. Briefly, C6 NPs were synthesized using an oil-in-water (o/w) single emulsion technique [10, 15, 41]. Carboxyl-terminated poly(lactic *co*-glycolic acid, PLGA) (0.55–0.75 dL/g, LACTEL^®^) was used to synthesize 100–200 mg batches. Coumarin 6 was dissolved in methylene chloride (DCM) overnight at a concentration of 15 μg C6 per mg of PLGA. The following day, the PLGA/C6/DCM solution was added dropwise to a 5% polyvinyl alcohol (PVA) solution of equal volume, vortexed and sonicated. The resulting NPs were hardened in 0.3% PVA during solvent evaporation for 3 h.

Unmodified NPs were washed after hardening, and centrifuged at 4 °C, 3 times in deionized water (diH_2_O) to remove residual solvent. NPs were frozen, lyophilized, and stored at − 20 °C until use. A similar protocol was followed to synthesize MPG (3177 Da, GenScript) and PEG (5000 Da, Nanocs Inc.) modified NPs, with the addition of avidin-palmitate (1 mg/mL) to the 5% PVA solution. Surface-modified NPs were collected after the first wash, and incubated for 30 min with biotinylated ligands at a molar ratio of 3:1 ligand:avidin in PBS. After the conjugation reaction, the NPs were washed two more times with diH_2_O centrifugation, frozen, and lyophilized. All NPs were stored at − 20 °C after synthesis.

### Nanoparticle characterization

NP characterization confirmed physical properties including NP diameter, morphology, and surface charge. First, scanning electron microscopy (SEM, Zeiss SUPRA 35VP) was utilized to verify NP morphology. Unhydrated NP diameters were measured using NIH ImageJ software. NP surface charges were characterized using a Malvern Zetasizer (Zetasizer Nano ZS90).

### Three-dimensional cell cultures

The human cervical carcinoma cell lines, SiHa, and CaSki (ATCC), were kindly provided by Dr. Alfred Jenson (University of Louisville), while the HeLa cell line was generously provided by Dr. Kenneth Palmer (University of Louisville). HeLa and SiHa cells were maintained in Minimum Essential Media (MEM) and CaSki cells were maintained in RPMI medium, both supplemented with 10% fetal bovine serum and 1% penicillin–streptomycin. All cells were kept in a humidified atmosphere of 5% CO_2_ at 37 °C, and were grown to 80% confluence prior to tumor spheroid formation.

### Liquid overlay tumor spheroid formation

**Fig. 7:**
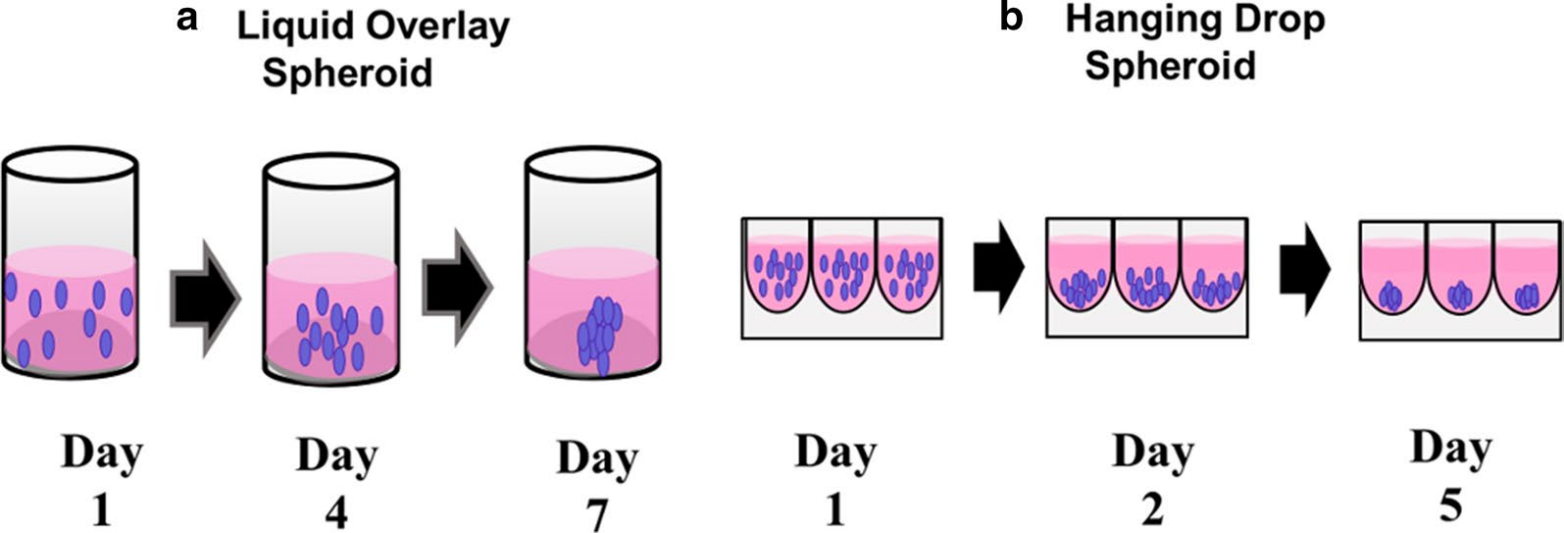
Schematic of spheroid formation techniques for **a** liquid overlay (LO) and **b** hanging drop (HD) spheroids, respectively representing larger, more irregularly-shaped and smaller, regularly-shaped avascular tissue

Liquid overlay spheroids were grown as previously described [12, 15, 46]. Briefly, to prevent spheroid adherence to the plate, 24-well tissue culture plates (#353047, Corning) were coated with a 1% (w/v) agarose gel 6 h prior to spheroid formation. After trypsinization, cells were collected and plated at a density of 100,000 cells per well containing 700 μL culture medium and lightly shaken (100 rpm) for 15 min on a reciprocating shaker. Following this, culture plates were transferred to an incubator and maintained at 37 °C and 5% CO_2_. Spheroids were grown for 7 days with culture media changes every 2 days (Fig. 7).

#### Hanging drop tumor spheroid formation

To form hanging drop spheroids, ultra-low attachment plates (#4515, Corning) were utilized. Briefly, cells were trypsinized after reaching 80–90% confluency and seeded at a density of 5000 cells per well in 100 μL culture medium. Care was taken to minimize pipette tip contact with ultra-low attachment plate well walls. Spheroids were allowed to form for 5 days under the cell culture conditions described above (Fig. 7).

#### Spheroid characterization

**Fig. 8:**
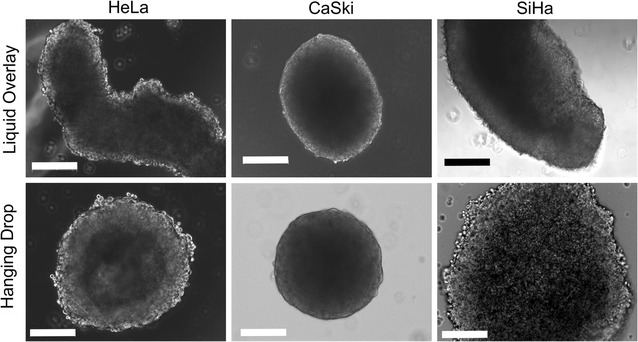
Typical morphologies of liquid overlay (LO) and hanging drop (HD) tumor spheroids, evaluated via bright field microscopy. Scale bar: 200 μm

Spheroid morphology was characterized prior to NP administration. Briefly, spheroids were removed from culture plates and placed on imaging dishes (P35G-0-14-C, MatTek) in 25 μL of culture medium to prevent drying. Spheroids were then imaged using an epifluorescent microscope (Axiovision 4, Zeiss) under transmitted light using a 10 × objective (Fig. 8).

### NP distribution

To assess NP distribution in tumor spheroids, four different NP formulations were evaluated: unmodified, PEG, MPG, and MPG/PEG co-treatment groups. Once spheroid formation was achieved using the hanging drop (5 days) and liquid overlay (7 days) techniques, fresh culture medium was added and spheroids were incubated with 10 μg/mL NPs for 1.5 h. For the NP co-treatment group, a half-dose of the MPG (5 μg/mL) and PEG (5 μg/mL) NPs were combined for a total NP dose of 10 μg/mL. After NP administration, both LO and HD individual tumor spheroids, were transferred to eppendorf tubes for fluorescent staining. Due to the differing spheroid sizes that result from the LO and HD methods, the total volume transferred was either 100 or 50 μL, for LO and HD spheroids, respectively. Once transferred, tumor spheroids were washed with 0.2 mL of 1X PBS and fixed with 0.2 mL of 4% paraformaldehyde for 10 min at RT. Subsequent to fixation, spheroids were treated with 0.2 mL of 1% Triton-X for 10 min at RT. Spheroids were subsequently washed twice with 0.2 mL of PBS, followed by treatment with 0.2 mL of 4 μg/mL Hoescht in 1% BSA PBS++ (containing CaCl_2_ and MgCl_2_) for 10 min at RT for nuclear staining. Finally, spheroids were washed in 0.2 mL of PBS and once in 0.2 mL of DI water. Spheroids were then transferred to imaging dishes (P35G-0-14-C, MatTek) suspended in 50 μL PBS.

NP uptake and distribution through tumor spheroids were assessed via confocal microscopy (LSM 710, Zeiss) and image analysis was performed using ImageJ software. The following laser settings: 4′ 6-diamidino (DAPI) and GFP were used to visualize Hoechst (blue, cell nuclei) and C6 (green, within NPs), respectively. A laser intensity of 2 and a gain of 600 were used for the DAPI/Hoechst channel, while a laser intensity of 5 and a gain of 500 were maintained for the GFP/C6 channel across experiments. Imaris x64 (v7.7.2, Bitplane) was utilized to generate 3D images from the composite z-stacks of the tumor spheroids. These 3D images were then rotated 90°, forming cross-sections of the tumor spheroids (Additional file 2: Figure S2). Using ImageJ, at least 3 representative samples were taken from the tumor cross-sections to evaluate NP distribution within the spheroids. NP penetration was quantified by plotting the mean fluorescence intensity (MFI) of each 3D optical reconstruction as a function of distance from the periphery of the spheroid towards the midplane. At least 8 fields of view were analyzed per sample, of which the averages and standard deviations are reported. NP internalization was then assessed by analyzing the area under the curve (AUC, MFI-μm) of the generated distribution profiles using a trapezoidal approximation in Excel. Statistical significance of NP treatment group penetration was determined using a one-way ANOVA post hoc Tukey test < 0.05.

## Additional files

**Additional file 1: Figure S1.** NP distribution represented as AUC for each NP treatment group as a function of tumor cell type (HeLa, CaSki, or SiHa), relative to spheroid type (LO, black and HD, gray). Values of all significant correlations for a particular treatment group, for each cell type relative to other cell types or relative to the same cell type in a different spheroid type are given with degree of significance indicated (* p < 0.01, ** p < 0.001, *** p < 0.0001, **** p < 0.00001). Error bars: average ± standard deviation (n = 3).

**Additional file 2: Figure S2.** Process to measure the NP distribution through the tumor spheroids via measurement of fluorescence intensity.

## Abbreviations

AUC: area under the curve
C6: Coumarin 6
CPP: cell penetrating peptide
DCM: methylene chloride
diH_2_O: deionized water
HD: hanging drop
HeLa: human cervical carcinoma
HPV: human papilloma virus
LO: liquid overlay
MEM: minimum essential media
MFI: mean fluorescence intensity
NP: nanoparticle
o/w: oil-in-water
PA-NHS: palmitic acid-*N*-hydroxysuccinimide ester
PBS: phosphate buffered saline
PEG: polyethylene glycol
PLGA: poly(lactic-*co*-glycolic) acid
PVA: polyvinyl alcohol
SEM: scanning electron microscopy
