# Supplementary figures and images for: Distribution of PLGA-modified nanoparticles in 3D cell culture models of hypo-vascularized tumor tissue
Source: J Nanobiotechnology. 2017 Oct 5;15:67. doi: 10.1186/s12951-017-0298-x (PMC5629750; doi:10.1186/s12951-017-0298-x)

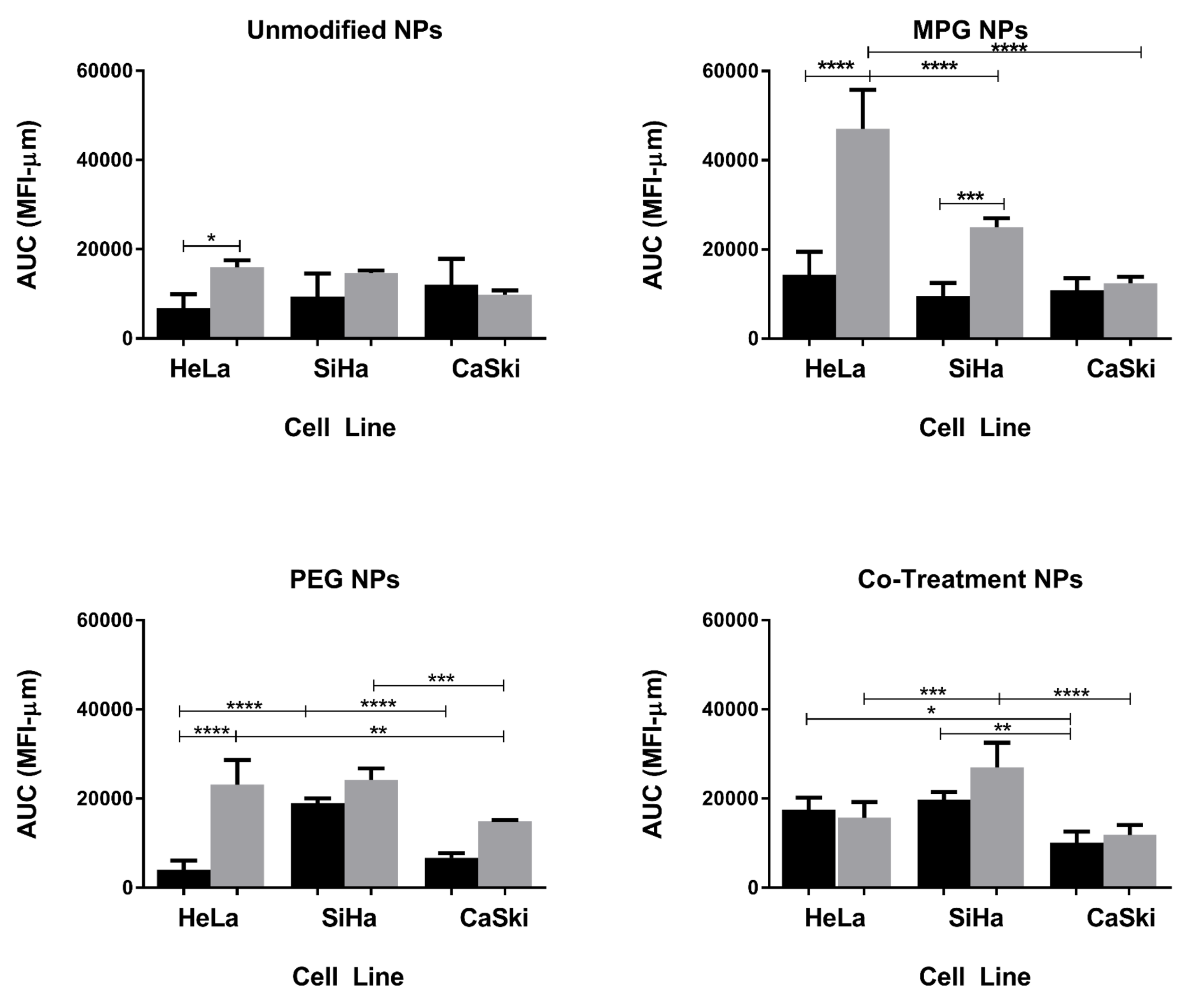

Supplement: Supplementary file 1 — Additional file 1: Figure S1. NP distribution represented as AUC for each NP treatment group as a function of tumor cell type (HeLa, CaSki, or SiHa), relative to spheroid type (LO, black and HD, gray). Values of all significant correlations for a particular treatment group, for each cell type relative to other cell types or relative to the same cell type in a different spheroid type are given with degree of significance indicated (* p < 0.01, ** p < 0.001, *** p < 0.0001, **** p < 0.00001). Error bars: average ± standard deviation (n = 3). [file 12951_2017_298_MOESM1_ESM.tif]

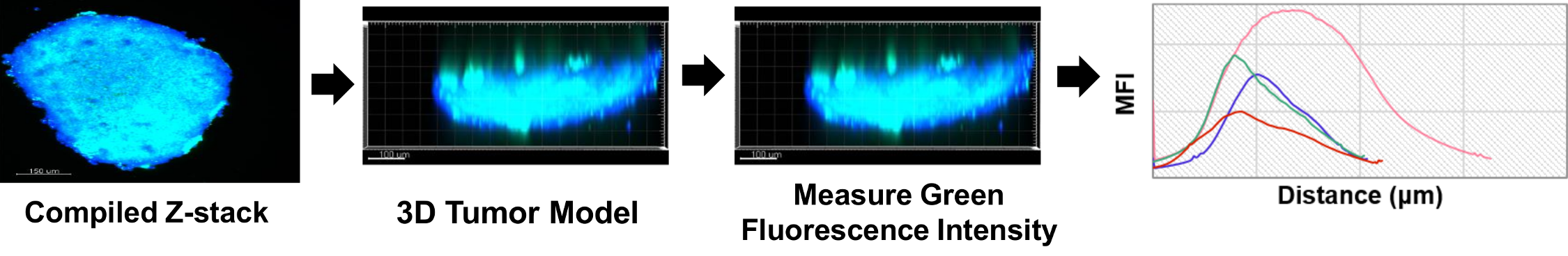

Supplement: Supplementary file 2 — Additional file 2: Figure S2. Process to measure the NP distribution through the tumor spheroids via measurement of fluorescence intensity. [file 12951_2017_298_MOESM2_ESM.tif]
